# Transdiagnostic Role of Glutamate and White Matter Damage in Neuropsychiatric Disorders: A Systematic Review

**DOI:** 10.1192/j.eurpsy.2022.440

**Published:** 2022-09-01

**Authors:** I. Luttenbacher, A. Philips, R. Kazemi, A. Hadipour, I. Sanghvi, J. Martinez, M. Adamson

**Affiliations:** 1 University of Amsterdam, Social And Behavioral Sciences, Amsterdam, Netherlands; 2 Veterans Affairs Palo Alto Health Care System, Rehabilitation Service, Palo Alto, United States of America; 3 Stanford University School of Medicine, Department Of Psychiatry & Behavioral Sciences, Stanford, United States of America; 4 University of Tehran, Department Of Psychology, Cognitive Lab, Tehran, Iran; 5 University of Tehran, Atieh Clinical Neuroscience Center, Tehran, Iran; 6 University of Messina, Department Of Cognitive Sciences, Messina, Italy; 7 University of Southern California, Department Of Neuroscience, Los Angeles, United States of America; 8 Palo Alto University, Department Of Psychology, Palo Alto, United States of America; 9 Stanford University School of Medicine, Department Of Neurosurgery, Stanford, United States of America

**Keywords:** White matter, Transdiagnostic, Neuropsychiatric Disorders, Glutamate

## Abstract

**Introduction:**

Neuropsychiatric disorders including Generalized Anxiety Disorder (GAD), Obsessive-Compulsive Disorder (OCD), Major Depressive Disorder (MDD), Bipolar Disorder (BD), and Schizophrenia (SZ) have been considered distinct categories of diseases despite their overlapping characteristics and symptomatology.

**Objectives:**

We aimed to provide an in-depth review elucidating the role of glutamate/Glx and white matter (WM) abnormalities from a transdiagnostic perspective.

**Methods:**

The PubMed online database was searched for studies published between 2010 and 2021. After careful screening, 399 studies were included.

**Results:**

The findings point to decreased levels of glutamate in the Anterior Cingulate Cortex in both SZ and BD, whereas Glx is elevated in the Hippocampus in SZ and MDD. With regard to WM abnormalities, the Corpus Callosum and superior Longitudinal Fascicle were the most consistently identified brain regions showing decreased fractional anisotropy (FA) across all the reviewed disorders, except GAD. Additionally, the Uncinate Fasciculus was found to be affected in all the reviewed disorders, except OCD. Decreased FA was also found in the inferior Longitudinal Fasciculus, inferior Fronto-Occipital Fasciculus, Thalamic Radiation, and Corona Radiata in SZ, BD, and MDD. Decreased FA in the Fornix and Corticospinal Tract were found in BD and SZ patients. The Cingulum and Anterior Limb of Internal Capsule exhibited decreased FA in MDD and SZ patients.

**Conclusions:**

The results suggest a gradual increase in severity from GAD to SZ defined by the number of brain regions with WM abnormality which may be partially caused by abnormal glutamate levels. WM damage could thus be considered a potential marker of some of the main neuropsychiatric disorders.

**Disclosure:**

No significant relationships.

